# Usefulness of preoperative coronary computed tomography angiography in high risk non-cardiovascular surgery old patients with unknown or suspected coronary artery disease

**DOI:** 10.1186/s12872-020-01731-7

**Published:** 2020-10-15

**Authors:** Xue-Ming Li, Zhong-Zhi Xu, Zhi-Peng Wen, Jiao Pei, Wei Dai, Huai-Ming Wang, Jing Reng, Peng Zhou, Guo-Hui Xu

**Affiliations:** 1 Department of Radiology, Sichuan Cancer Hospital and Institute, Sichuan Cancer Center, School of Medicine, University of Electronic Science and Technology of China, 55# Lan 4 RenMing Road (South), Chengdu, 610041 Sichuan Province China; 2 Department of Statistics, Sichuan Cancer Hospital and Institute, Sichuan Cancer Center, School of Medicine, University of Electronic Science and Technology of China, 55# Lan 4 RenMing Road (South), Chengdu, 610041 Sichuan Province China; 3 Department of Thoracic Surgery, Sichuan Cancer Hospital and Institute, Sichuan Cancer Center, School of Medicine, University of Electronic Science and Technology of China, 55# Lan 4 RenMing Road (South), Chengdu, 610041 Sichuan Province China; 4 Department of Anesthesiology, Sichuan Cancer Hospital and Institute, Sichuan Cancer Center, School of Medicine, University of Electronic Science and Technology of China, 55# Lan 4 RenMing Road (South), Chengdu, 610041 Sichuan Province China

**Keywords:** Computed tomography, Angiography, Coronary artery disease, Non-cardiovascular, Surgery, Perioperative period

## Abstract

**Background:**

Cumulative evidence has shown that the non-invasive modality of coronary computed tomography angiography (CCTA) has evolved as an alternative to invasive coronary angiography, which can be used to quantify plaque burden and stenosis and identify vulnerable plaque, assisting in diagnosis, prognosis and treatment. With the increasing elderly population, many patients scheduled for non-cardiovascular surgery may have concomitant coronary artery disease (CAD). The aim of this study was to investigate the usefulness of preoperative CCTA to rule out or detect significant CAD in this cohort of patients and the impact of CCTA results to clinical decision-making.

**Methods:**

841 older patients (age 69.5 ± 5.8 years, 74.6% males) with high risk non-cardiovascular surgery including 771 patients with unknown CAD and 70 patients with suspected CAD who underwent preoperative CCTA were retrospectively enrolled. Multivariate logistic regression analysis was performed to determine predictors of significant CAD and the event of cancelling scheduled surgery in patients with significant CAD.

**Results:**

677 (80.5%) patients had non-significant CAD and 164 (19.5%) patients had significant CAD. Single-, 2-, and 3- vessel disease was found in 103 (12.2%), 45 (5.4%) and 16 (1.9%) patients, respectively. Multivariate analysis demonstrated that positive ECG analysis and Agatston score were independently associated with significant CAD, and the optimal cutoff of Agatston score was 195.9. The event of cancelling scheduled surgery was increased consistently according to the severity of stenosis and number of obstructive major coronary artery. Multivariate analysis showed that the degree of stenosis was the only independent predictor for cancelling scheduled surgery. In addition, medication using at perioperative period increased consistently according to the severity of stenosis.

**Conclusions:**

In older patients referred for high risk non-cardiovascular surgery, preoperative CCTA was useful to rule out or detect significant CAD and subsequently influence patient disposal. However, it might be unnecessary for patients with negative ECG and low Agatston score.

*Trial registration* Retrospectively registered.

## Background

A major predisposing factor in the pathogenesis of perioperative cardiovascular events is the presence of ischemic heart disease, whether diagnosed or previously unknown [1]. Atherosclerosis is the main pathological disorder responsible for the development of ischemic heart disease. With the increasing elderly population, many patients scheduled for non-cardiovascular surgery may have concomitant coronary artery disease (CAD) [1]. Therefore, identifying patients at risk before operation is sensible.

Invasive coronary angiography (ICA) is a well-established diagnostic procedure, but it is rarely recommended to assess the risk of non-cardiovascular surgery in routine tests unless the patient has an independent indication for angiography [1, 2]. In addition, it has high radiation-exposure and may cause unnecessary and unpredictable delay in an already planned surgical intervention [2]. Coronary computed tomography angiography (CCTA) has recently emerged as a fast, noninvasive and robust imaging modality for the visualization of coronary arteries with high resolution which can quantify plaque burden and severity of CAD without physiological or pharmacological stress, and it also has obvious advantages of low radiation, being less invasive and cheaper. Moreover, studies have indicated that CCTA can reliably replace ICA as a screening tool before valve operation [3–5]. As to the risk stratification of preoperative CCTA, there was no definitive recommendations in previous ACC/AHA and ESC/ESA guidelines [1, 2], and it was not recommended in the recent Canadian Cardiovascular Society guidelines [6].

The aim of this study was to investigate the usefulness of preoperative CCTA to rule out or detect significant CAD in this cohort of patients and the impact of CCTA results to clinical decision-making, thus increasing our understanding of perioperative management.

## Methods

### Study subjects

**Fig. 1:**
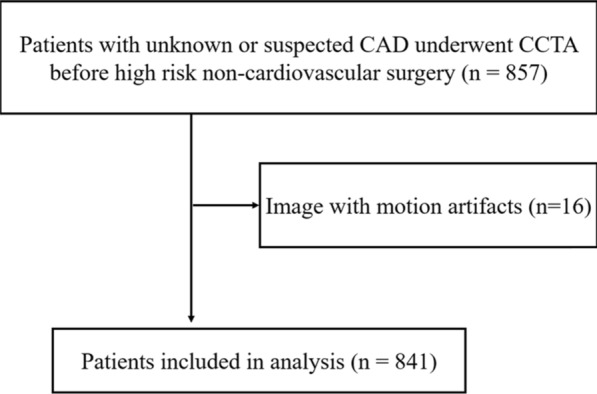
Flow diagram of the study patients. *CAD* coronary artery disease, *CCTA* coronary computed tomography angiography

This study was approved by the local institutional review board and informed consent was waived for all subjects because of the study’s retrospective design. 857 older patients (age ≥ 60 yeas) with elective high-risk type of non-cardiovascular surgery [2] who underwent preoperative CCTA for screening of CAD were enrolled from September 2012 to June 2019 in our institution. Patients with severe arrhythmia (e.g. atrial fibrillation), iodine allergy, renal dysfunction (glomerular filtration rate < 30 mL/min/1.7m^2^) and a left ventricular ejection fraction of less than 40% were not eligible for CCTA. Sixteen patients with motion artifacts were excluded, and 841 patients (mean age 69.5 ± 5.8 years, 74.6% men) were finally included in the analysis (Fig. 1). Patients were stratified as unknown or suspected CAD, and the suspected CAD was defined when patients had clinical symptoms of angina or dyspnea on exertion, positive electrocardiogram (ECG) suggesting myocardial ischemia, multiple coronary risk factors such as hypertension, diabetes mellitus, hyperlipidemia, current smoking and stroke. The major clinical indications for non-cardiovascular surgery were lung tumors (324 patients), esophageal and gastric carcinoma (453 patients) and mediastinal tumor (64 patients).

### CT protocol

CCTA examinations were performed on a wide detector 256-slice CT scanner (Brilliance iCT; Philips Healthcare, Cleveland, OH, USA) in all patients. Oral betablocker (25–50 mg) was administered 60 min prior to the examination if necessary, and the target heart rate (HR) was under 90 beats per minute (bpm). The scanning scope was from the tracheal bifurcation to 20 mm below the inferior cardiac apex.

Firstly, a native scan without contrast medium was performed to calculate the total coronary calcium burden using Agatston method. This scan was prospectively triggered at 70% or 75% of the R-R interval and performed using the following parameters: tube voltage 120 kV, tube current 550 mAs, rotation time 0.27 s, slice thickness 2.5 mm and reconstruction interval 2.5 mm. Next, a volume data set was acquired after antecubital venous injection of 1 to 1.5 mL/kg contrast medium (Ultravist 370, Bayer Healthcare, Berlin, Germany) at a flow rate of 5 mL/s followed by 30 ml saline solution using a 20-gauge needle with double tube high-pressure syringe (BolusPro, Philips Healthcare, Cleveland, Ohio, USA). Contrast material injection time was determined by the bolus-tracking technique in the ascending aorta [trigger threshold 100–120 Hounsfield units (HU)], and ECG gated data acquisition was initiated 6 s after triggering. The scanning parameters were as follows: tube voltage 100 kV, tube current 400–500 mAs, detector collimation 128 × 0.625 mm, rotation time 0.27 s, pitch 0.18, slice thickness 0.9 mm and reconstruction interval 0.45 mm.

### Data analysis

All the images were transferred to an external workstation (Cardiac Viewer and Comprehensive Cardiac Analysis, Extended Brilliance Workspace (Version 4.0); Philips Healthcare, Cleveland, Ohio, USA) and coronary artery stenosis was interpreted with maximum intensity projection (MIP), multiplanar reconstruction (MPR), curvature plane reconstruction (CPR) and volume rendering (VR). Two professional radiologists with more than 5-years of cardiac CT experience analyzed all the images in consensus, and a third reader provided consensus in cases of disagreement. We firstly divided patients into no CAD, non-obstructive CAD and obstructive CAD, then patients were stratified based on the presence and severity of CAD into non-significant CAD with no CAD or nonobstructive CAD (1–49%) and significant/obstructive CAD with moderate (50–69%) and/or severe (≥ 70%) stenosis. No CAD was considered when there is no plaque detected in any of the major epicardial arteries. Single-, 2-, and 3-vessel disease was defined based on the number of obstructive major epicardial arteries.

In addition, we retrospectively and carefully investigated the clinical data on electronic medical records to evaluate the cardiovascular factors, ECG, blood tests, echocardiography, revised cardiac risk index (RCRI) and clinical decision-making. Routine ECG were performed prior to CCTA, ST-segment analysis was considered positive if horizontal or down-sloping ST-segment depression ≥ 1 mm was found in ≥ 2 consecutive leads. The clinical perioperative cardiac risk of RCRI was assessed as the number of following perioperative risk factors, such as high-risk surgery, history of ischemic heart disease, cerebrovascular disease, pulmonary edema, insulin-dependent diabetes mellitus, and serum creatinine > 2.0 mg/dL [7]. The impact of CCTA results on clinical decision-making was evaluated by our multidisciplinary tumor board, including whether cancel scheduled surgery for the reason of significant CAD and the medication using in the perioperative period.

### Statistical analysis

Statistical analysis was performed using SPSS software (version 17.0 for windows; SPSS, Chicago, IL, USA). Continuous variables were expressed as mean ± standard deviation (SD) or as median (25th–75th percentile range) as appropriate. Nominal variables were expressed as frequency and percentages. For continuous variables, differences were assessed with ANOVA or Kruskal–Wallis H (K) analysis, as appropriate. And for categorical variables, differences in proportions were analyzed using the chi-square or Fisher exact test, as appropriate. Univariate and multivariate logistic regression analysis were performed to evaluate which parameters were independently associated with the diagnosis of CAD or clinical decision-making. All variables with clinical significance and/or *p* value < 0.1 in the univariate analyses were introduced to further multivariate analysis. In addition, receiver operating characteristic curve (ROC) analysis was carried out to identify the patients who would have significant CAD at CCTA. A two-tailed *p* < 0.05 was considered statistically significant.

## Results

### Patient characteristics

Patient characteristics of the entire trial cohort (the 841 study subjects) and those stratified by CT coronary categories are presented in Table 1. With increasing severity of CT-based CAD categories, patients were more likely to be men, suspected of CAD, had more positive ECG results, Agatston score and cardiac risk factors including smoking, diabetes and hypertension; while the heart rate and LVEF were not significantly different at the baseline. Among the 841 patients, 771 patients had unknown CAD and 70 patients were suspected for CAD. The patients with suspected CAD had higher rate of significant CAD than those with unknown CAD (38.6% vs 17.8%, *p* < 0.001).

**Table 1:** Patient characteristics of the entire trial cohort and those stratified by CT CAD categories

Variables	Total cohort	No CAD	Nonobstructive CAD	Obstructive CAD	*p* value
	n = 841	n = 485	n = 192	n = 164	n = 841
Age (years)	69.5 ± 5.8	68.4 ± 5.5	70.6 ± 6.3	71.3 ± 5.4	< 0.001
Male	627 (74.6)	334 (68.9)	155 (80.7)	138 (84.1)	< 0.001
Suspected CAD	70 (8.3)	28 (5.8)	15 (7.8)	27 (16.5)	0.001
Positive ECG	110 (13.1)	48 (10.0)	17 (8.9)	45 (27.4)	< 0.001
HR (beats/min)	73.6 ± 25.7	74.0 ± 30.9	72.8 ± 17.2	73.3 ± 13.8	0.855
LVEF (%)	66.6 ± 7.1	66.5 ± 6.7	67.0 ± 6.9	66.4 ± 8.3	0.655
Median Agatston score^†^		0	71 (17.4, 190.2)	348.4 (124.3, 789.9)	< 0.001
Risk factors					
Smoking	401 (47.7)	210 (43.3)	100 (52.1)	91 (55.5)	0.003
Diabetes mellitus	85 (10.1)	38 (7.8)	21 (10.9)	26 (15.9)	0.004
Hypertension	293 (34.8)	138 (28.5)	73 (38.0)	82 (50.0)	< 0.001
Hyperlipidemia	248 (29.5)	137 (28.2)	57 (29.7)	54 (32.9)	0.272
Stroke	26 (3.1)	12 (2.5)	5 (2.6)	9 (5.5)	0.101
Medication use at perioperative period					
Statins	80 (9.5)	16 (3.3)	11 (5.7)	53(32.3)	< 0.001
ACEi or ARB	66 (7.8)	27 (5.6)	17 (8.9)	22 (13.4)	0.001
Calcium channel blockers	195 (23.2)	91 (18.8)	52 (27.1)	52 (31.7)	< 0.001
Beta-blocker	124 (14.7)	53 (10.9)	26 (13.5)	45 (27.4)	< 0.001
Diuretics	288 (34.2)	148 (30.5)	81 (42.2)	59 (36.0)	0.06
Nitrate agent	184 (21.9)	86 (17.7)	45 (23.4)	53 (32.3)	< 0.001

Unless otherwise indicated, values are mean ± standard deviations (SD) or n (%)

*CT* computed tomography, *CAD* coronary artery disease, *ECG* electrocardiography, *HR* heart rate, *LVEF* left ventricular ejection fraction, *ACEi* angiotensin converting enzyme inhibitor, *ARB* angiotensin II receptor blocker

*p* value represents comparison among no CAD, non-obstructive and obstructive CAD

^†^Data in parentheses are interquartile ranges

### Cardiac CT findings

**Fig. 2:**
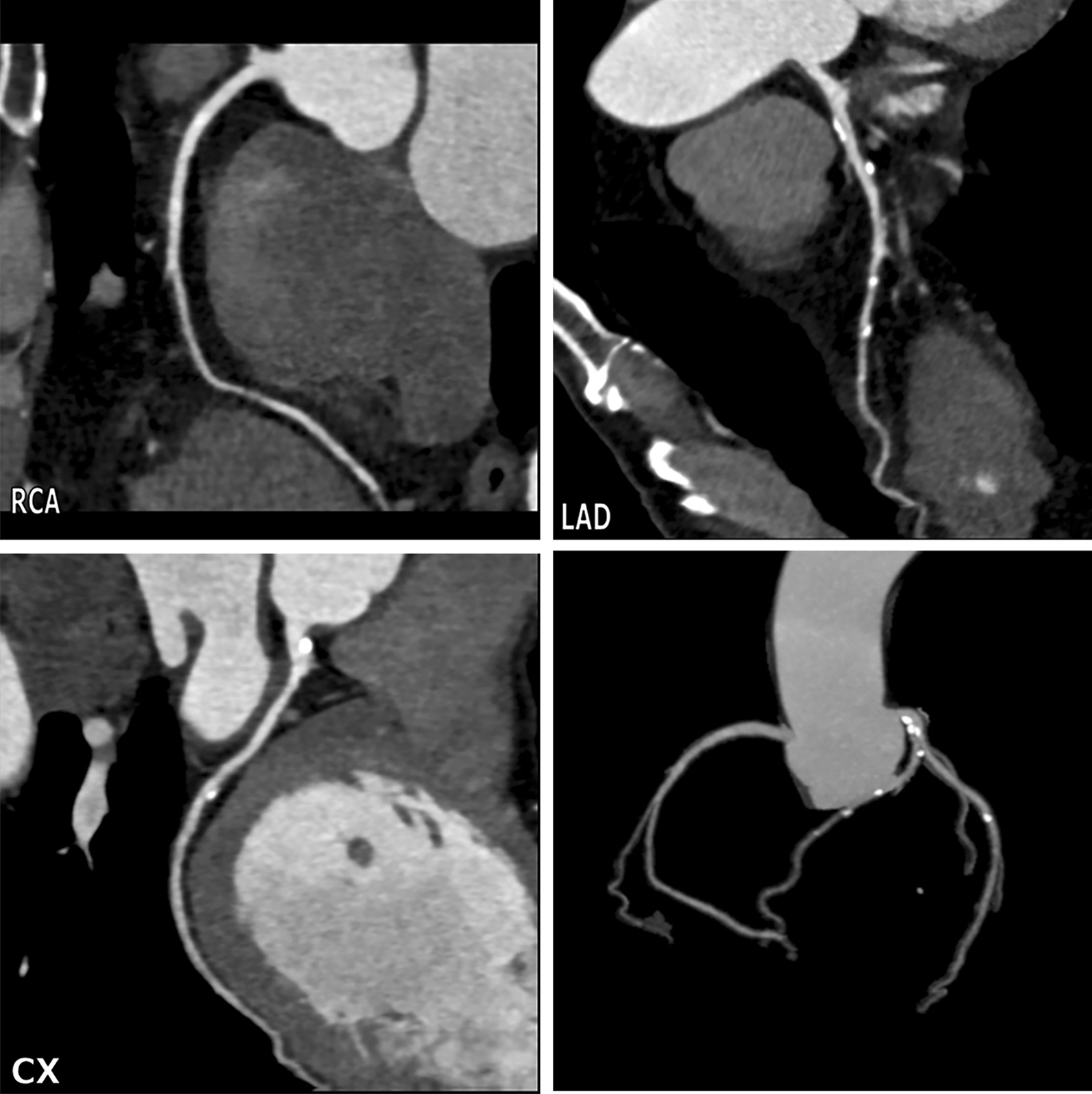
An example of mild stenosis in 56-year old asymptomatic man with negative ECG analysis. Multiple calcified plaque with mild stenosis in the left main artery, left anterior descending coronary artery (LAD) and left circumflex coronary artery (CX). The right coronary artery (RCA) is normal

**Fig. 3:**
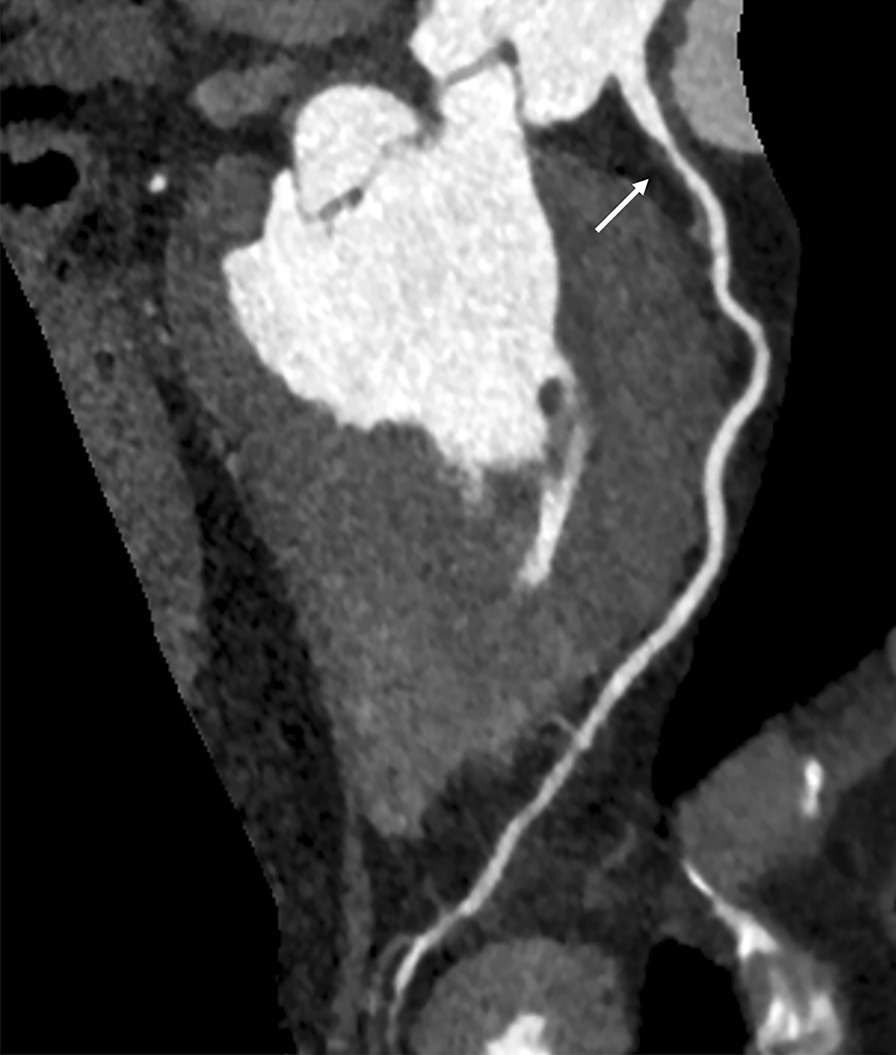
An example of moderate stenosis in 68-year old asymptomatic women with negative ECG analysis. Non-calcified plaque with moderate stenosis in the proximal segment of left anterior descending coronary artery (arrow)

**Fig. 4:**
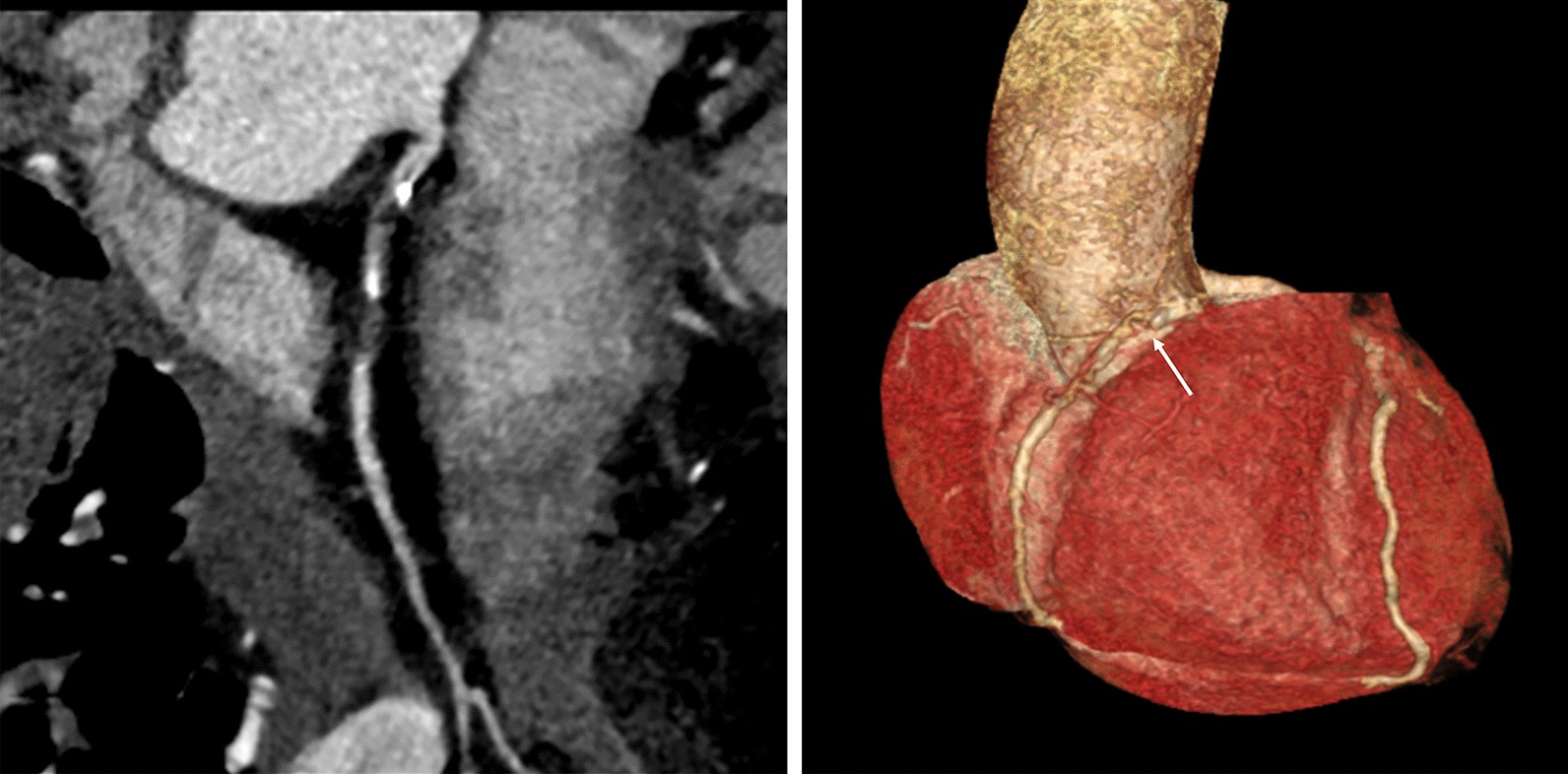
An example of severe stenosis in 73-year old man with chest pain and positive ECG analysis. Mixed plaque with severe stenosis in the proximal segment of the right coronary artery (arrow)

Table 2 shows the coronary categories as determined by CT. In total, 677 (80.5%) patients had non-significant CAD and 164 (19.5%) patients had significant CAD. Of the patients with non-significant CAD, 485 (57.7%) patients were normal and 192 (22.8%) patients showed mild stenosis (Fig. 2). Of the patients with significant CAD, 78 (9.3%) patients had moderate stenosis (Fig. 3) and 86 (10.2%) patients had severe stenosis (Fig. 4). In addition, single-, 2-, and 3- vessel disease was found in 103 (12.2%), 45 (5.4%), and 16 (1.9%) patients, respectively (Table 2); and 61 (7.3%) patients showed multi-vessel disease (≥ 2 branches).

**Table 2:** Coronary categories as determined by CT and the events of abandoned surgery for the reason of significant CAD

	Frequency (n, %)	Event (n, %)	*p* value
Maximal stenosis of any coronary artery			
Non-significant CAD			
No-CAD	485 (57.7%)	0 (0)	
1–49%	192 (22.8%)	0 (0)	
Significant CAD			0.008
50–69%	78 (9.3%)	30 (38.5%)	
≥ 70%	86 (10.2%)	52 (60.5%)	
Number of obstructive major coronary artery			
Maximal stenosis < 50%	677 (80.5%)	0 (0)	
1-vessel disease	103 (12.2%)	46 (44.7%)	0.068^†^
2-vessel disease	45 (5.4%)	24 (53.3)	
3-vessel disease	16 (1.9%)	12 (75.0%)	
Multi-vessel disease	61 (7.3%)	36 (59.0%)	0.106^‡^

*CAD* coronary artery disease

^†^Event compared among 1, 2 and 3-vessel disease

^‡^Event compared between 1-vessel disease

**Fig. 5:**
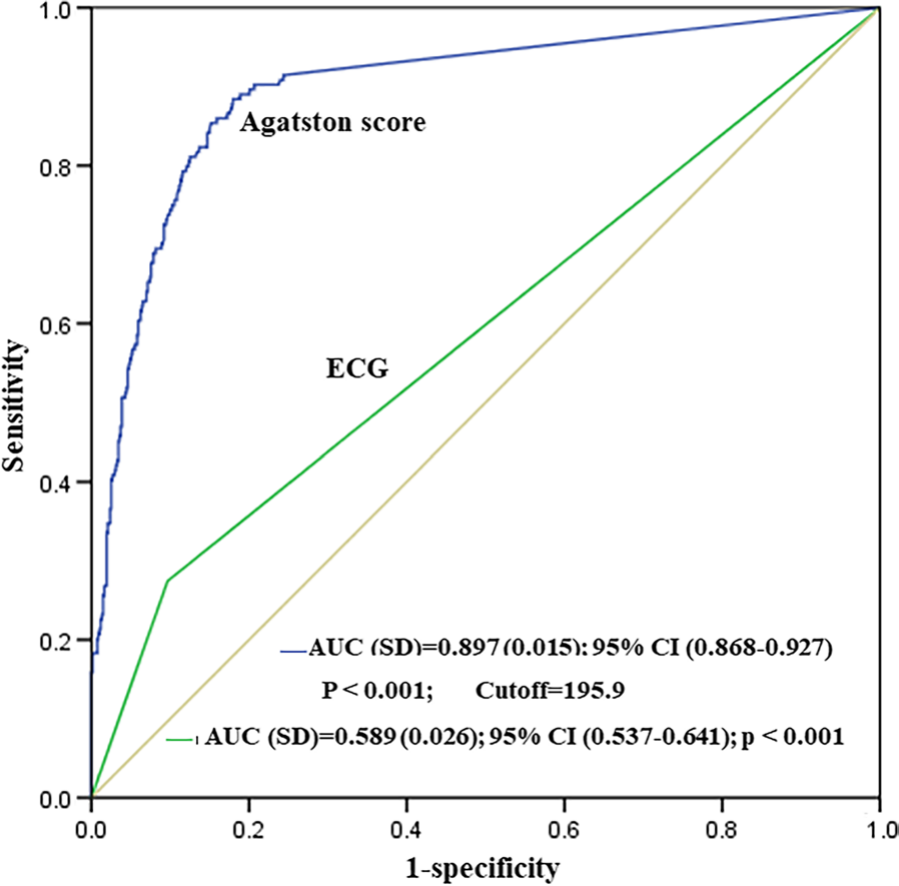
Receiver operating characteristic curve for models created to assess the ability of Agatston score and positive ECG analysis to diagnose significant coronary artery disease. *AUC* area under the receiver operating characteristic curve, *CI* confidence interval, *SD* standard deviation

The univariate and multivariate logistic regression analyses for the association between significant CAD and associated factors are shown in Table 3, which demonstrated that age, diabetes mellitus, positive ECG analysis and Agatston score were independently related with the diagnosis of significant CAD. ROC analysis for the diagnosis of significant CAD was presented in Fig. 5. The area under ROC curve (AUC) for Agatston score and positive ECG analysis for the diagnosis of significant CAD was 0.897 {95% confidence interval (CI) of AUC = 0.868–0.927} and 0.589 {95% confidence interval (CI) of AUC = 0.537–0.641}, respectively. The cutoff of Agatston score with the highest sensitivity and specificity (62.8% and 93.0%, respectively) was195.9.

**Table 3:** Univariate and multivariate logistic regression analysis for the detection of significant CAD

Variables	Univariate analysis	*p*	Multivariate analysis	*p*
	OR (95% CI)		OR (95% CI)	
Age (years)	1.066 (1.036–1.097)	< 0.001	1.054 (1.014–1.095)	0.008
Male	2.041 (1.299–3.205)	0.002	1.720 (0.909–3.255)	0.095
Suspected CAD	2.906 (1.735–5.866)	< 0.001	1.817 (0.912–3.617)	0.089
Positive ECG	3.537 (2.306–5.426)	< 0.001	2.958 (1.701–5.146)	< 0.001
HR (beats/min)	0.999 (0.992–1.007)	0.857		
LVEF (%)	0.996 (0.972–1.021)	0.739		
Agatston score	1.006 (1.005–1.007)	< 0.001	1.005 (1.004–1.006)	< 0.001
Risk factors				
Smoking	1.476 (1.047–2.080)	0.026	0.855 (0.509–1.435)	0.552
Diabetes mellitus	1.973 (1.201–3.244)	0.007	1.969 (1.026–3.781)	0.042
Hypertension	2.209 (1.562–3.123)	< 0.001	1.338 (0.843–2.124)	0.216
Hyperlipidemia	1.222 (0.848–1.762)	0.282	1.439 (0.890–2.324)	0.137
Stroke	2.241 (0.980–5.121)	0.056	0.971 (0.292–3.226)	0.961

*OR* odds ratio, *CI* confidence interval, *CAD* coronary artery disease, *ECG* electrocardiography, *HR* heart rate, *LVEF* left ventricular ejection fraction

### CCTA results on clinical decision-making

There were 12 patients undergo an ICA after CCTA, 5 patients undergo coronary revascularization and one undergo surgical intervention after revascularization. The event of cancelling scheduled surgery increased consistently according to the severity of stenosis or the number of obstructive major coronary artery (Table 2). In patients with significant CAD, 82 patients including 30 (38.5%) patients with moderate stenosis and 52 (60.5%) patients with severe stenosis were cancelled for this reason (*p* = 0.008). In addition, scheduled surgery was cancelled in 46 (44.7%), 24 (55.3%) and 12 (75.0%) patients with single-, 2-, and 3- vessel disease, respectively. The univariate and multivariate logistic regression analyses for the association between the event of abandoned surgery and associated factors in patients with significant CAD were showed in Table 4, which demonstrated that degree of stenosis was independently related with the cancelling scheduled surgery.

**Table 4:** Univariate and multivariate logistic regression analysis for the event of abandoned surgery in patients with significant CAD

Variables	Univariate analysis	*p*	Multivariate analysis	*p*
	OR (95% CI)		OR (95% CI)	
Age	0.999 (0.943–1.057)	0.965		
Male Sex	1.201 (0.518–2.782)	0.669		
HR (beats/min)	1.019 (0.994–1.045)	0.130		
LVEF (%)	0.962 (0.922–1.004)	0.073	0.962 (0.920–1.007)	0.095
Agatston score	1.000 (1.000–1.001)	0.469	1.000 (0.999–1.000)	0.491
RCRI	1.445 (0.865–2.414)	0.160	1.331 (0.756–2.345)	0.322
Number of obstructive vessels	1.713 (1.059–2.770)	0.028	1.235 (0.645–2.364)	0.524
Degree of stenosis	2.447 (1.305–4.587)	0.005	2.543 (1.199–5.393)	0.015

*OR* odds ratio, *CI* confidence interval, *CAD* coronary artery disease; *HR* heart rate, *LVEF* left ventricular ejection fraction, *RCRI* revised cardiac risk index

In addition, medication using including statins, angiotensin converting enzyme inhibitor or angiotensin II receptor blocker (ACEI/ARB), calcium channel blockers, Beta-blocker of metoprolol tartrate and nitrate agent increased consistently according to the severity of stenosis (Table 1). Patients with multi-vessel disease had more statins using than those with one-vessel disease (42.6% vs 26.2%, *p* = 0.038).

## Discussion

The main findings of this study were that (1) preoperative CCTA can rule out or detect significant CAD and demonstrate the severity of disease in older patients referred for high risk non-cardiovascular surgery; (2) positive ECG analysis and Agatston score were independently associated with significant CAD; (3) Cancelling scheduled surgery and medication using increased consistently according to the severity of CAD detected by CCTA.

The aging population is increasing worldwide, and the concomitant coronary ischemic disease are not uncommon in the elderly [8]. There is an increasing number of patients with known or unknown CAD undergoing non-cardiovascular surgery, which represents a current problem in the clinical practice. Perioperative major cardiac events (PMCE), such as acute myocardial infarction, pulmonary edema, or primary cardiac death are often silent but the leading causes of death in perioperative period [1, 9, 10]. The precise mechanism is unclear, but stress induced rupture of vulnerable plaque and perioperative myocardial ischemia secondary to the imbalance between demand and oxygen availability are believed to play critical roles [11, 12].

To prevent the PMEC, cardiac assessment is of particular importance with documented or suspected CAD and in all patients undergoing surgery. Several noninvasive techniques have been suggested to identify patients who are at an elevated risk for noncardiac surgery, such as exercise ECG testing, stress echocardiography and stress myocardial perfusion imaging [1]. For the detection of myocardial ischemia, cardiac stress tests depend on the stress induced increase of myocardial oxygen demand or hyperemia [13]. However, it may not be tolerated or not be applied optimally in a considerable number of patients, such as patients with poor general condition, patients with contraindication to pharmacological agents (e.g., AV block or asthma), left ventricular dysfunction, or left bundle branch block [14]. Furthermore, it may be risk for stress test in patients with high-risk coronary anatomy (e.g., left main disease or multivessel disease involving proximal left anterior descending artery), and revascularization would be prioritized during elective noncardiac surgery [15]. In addition, it has a poor positive predictive value for perioperative cardiac events. Previous studies showed that CCTA can be used to exclude or find CAD with high sensitivity and specificity in symptomatic patients with low to intermediate pretest risk [16, 17]. In addition, it does not need any induction of cardiac stress. Therefore, CCTA can be performed when other noninvasive modalities are not adequate or contraindicated.

It seems appropriate to perform CCTA for patients scheduled to noncardiac surgery, whereas it was not regarded as standard clinical practice in current international guidelines [6]. In our study, 19.5% of patients have significant CAD after diagnostic coronary CT angiographic examination, however, most of them did not undergo ICA. Significant CAD may be over diagnosed in some of our patients due to severe calcification, leading to the overestimated cardiac risk, which was in consistent with previous study [18]. In the clinical practice of noninvasive preoperative screening for CAD, it is more concerned about avoiding underdiagnosis of CAD rather than overdiagnosis. Although ICA is not routinely recommended for risk stratification in non-cardiovascular surgery patients, in patients with acute cardiac conditions and high-risk ischemia with noninvasive stress testing, ICA and revascularization is recommended [1, 2, 19]. Further study should be implemented to focus on the question of whether all patients with significant coronary stenosis on CCTA should be evaluated with ICA. The multivariate analysis of our study showed that positive ECG analysis and Agatston score were independently associated with the diagnosis of significant CAD. The optimal cutoff of Agatston score was 195.9. Therefore, patients are at low risk of significant CAD and many CCTA examinations may be avoid if they have negative ECG and Agatston score under this cutoff.

Preoperative risk stratification provides a unique opportunity for the clinician not only to predict the short-term risk for a particular patient but also to estimate late cardiac events. Some studies have developed several perioperative risk prediction modules for perioperative risk stratification [2, 7]; and all of them emphasize that CAD is a major cause of mortality and morbidity. As a noninvasive and robust visualization tool for coronary artery anomalies, coronary artery stenosis and plaques, CCTA has shown unique value in predicting postoperative cardiovascular events and risk stratification for noncardiac surgeries [18, 20, 21]. Ji-won Hwang suggested that addition of CCTA to clinical risk improved perioperative risk stratification in patients undergoing noncardiac surgery [20]. Absence of significant CCTA findings conferred low PMCE risk with high specificity and negative predictive value regardless of clinical risk. Both the severity and extent of CAD were significantly associated with the risk of PMCE [22]. Significant stenosis and multivessel CAD were considered as significant indicators for postoperative events in addition to the RCRI [21], and an indicator of coronary revascularization [23]. A recent study showed that peri-operative risk may be refined further by employing nuclear myocardial perfusion imaging in patients with significant CAD on CCTA [24]. In addition, some studies suggest that plaque composition is associated with the clinical consequences of CAD. Noncalcified plaques are more prone to sudden plaque rupture, leading to acute ischemic coronary syndromes [25]. Noncalcified and mixed plaques may have poorer long-term clinical outcomes than calcified plaques [26]. Our results showed that scheduled surgery was cancelled to avoid PMCE in half of the significant CAD patients, and the event of abandoning planned surgery increased consistently according to the severity of stenosis or the number of obstructive major coronary artery.

Optimal perioperative medication use must be focused on the stabilizing of plaques, reducing the risk of perioperative myocardial ischemia and improving survival. Aspirin and statins may be benefit for patients with increased perioperative cardiac risk [27, 28], and discontinuation of them increased the risk of acute coronary syndrome especially in patients with stable ischemic heart disease [29, 30]. Perioperative beta blockers use was associated with a reduction in cardiac events, and it is recommended in patients already receiving this medication, especially for high risk surgery, however, they are not indicted in low risk surgery [1, 19]. Our study indicated that medication using including statins, antiplatelet agents, angiotensin converting enzyme inhibitor or angiotensin II receptor blocker (ACEI/ARB), calcium channel blockers, beta-blockers and nitrate agent at perioperative period increased consistently according to the severity of stenosis, and patients with multi-vessel disease have more statins using than those with one-vessel disease. We think that the CCTA results may lead treating more patients with medications. However, further study is needed to investigate the true benefit of perioperative medication use based on the CCTA results.

Several limitations in this study merit comment. First, this is an observational single-center study with potential center-specific bias, further multi-center studies are required to validate the present findings. Second, most of the patients did not undergo ICA despite they were diagnosed as significant CAD on CCTA which may be over diagnosed because of severe coronary calcification. Further studies with the aim of eliminating calcium blooming artifacts is of utmost importance for the success of CCTA. Third, renal insufficiency and allergy to contrast agents are contraindications for CCTA and radiation associated with CCTA is an issue of concern. Strategies with reduction of tube current or voltage and prospectively triggered acquisition are used to limit radiation dose. In the meantime, the introduction of new generation CT scanners can reduce radiation exposure, dramatically shorten scanning times and improve image quality. Finally, although we did not estimate the prognosis of patients using CCTA as a screening tool, this will be investigated in our future study.

## Conclusions

In older patients referred for high risk non-cardiovascular surgery, preoperative CCTA was useful to rule out or detect significant CAD, characterize the features of CAD, stratify the risk, and subsequently influence patient disposal. In addition, many CCTA examinations might be avoid in this cohort of patients if they have negative ECG results and low Agatston score.

## Data Availability

The datasets used and/or analyzed during the current study are available from the corresponding author on reasonable request.

## Abbreviations

CAD: Coronary artery disease
CTCA: Computed tomography coronary angiography
ICA: Invasive coronary angiography
AUC: Area under the receiver operating characteristic curve
